# Developing a Health Education Program for the Prevention and Control of Infectious Diseases Culturally Adapted to Ethnic and Rural Communities: Co-Design Study Using Participatory Audiovisual Methods

**DOI:** 10.2196/65116

**Published:** 2025-08-29

**Authors:** Martha Milena Bautista-Gomez, Laura Sofia Zuluaga Gutierrez

**Affiliations:** 1 Centro Internacional de Entrenamiento e Investigaciones Médicas (CIDEIM) Universidad Icesi Cali Colombia

**Keywords:** co-design of health interventions, participatory audiovisual methods, cultural adaptation, infectious diseases, health education program, ethnic communities

## Abstract

**Background:**

Infectious diseases disproportionately affect rural and ethnic communities in Colombia, where structural inequalities such as limited access to health care, poor sanitation, and scarce health education worsen their effects. Education is essential for preventing and controlling infectious diseases, fostering awareness of healthy behaviors, and empowering communities with the knowledge and skills to manage their health. Participatory and co-design methods strengthen educational programs by ensuring cultural relevance, enhancing knowledge retention, and promoting sustainable community interventions.

**Objective:**

This study aims to describe the co-design process and evaluate the capacity building of an education program for the prevention and control of infectious diseases using participatory audiovisual methods culturally adapted to ethnic communities and rural contexts in Colombia.

**Methods:**

A qualitative case study approach was used. 15 community leaders contributed to the program’s design, implementation, and evaluation. Nominal groups and a participatory social diagnosis identified key topics, while theoretical-practical sessions with visual methods guided the cocreation of workshops and audiovisual materials. Evaluation combined qualitative analysis of participants’ perceptions and quantitative assessment of knowledge acquisition. Qualitative data were coded through content analysis, while multiple-choice questionnaires (initial and final) categorized knowledge acquisition into 3 levels (low, medium, and high), with percentage distributions used for comparative analysis.

**Results:**

The co-design process resulted in 12 theoretical and practical workshops in infectious diseases and 3 audiovisual products: an animation about malaria, a comic book about cutaneous leishmaniasis, and a puppet show about tuberculosis. The quantitative evaluation applied to the 15 participants revealed substantial improvements, with the proportion that achieved excellent scores in pedagogy increasing from 40% (6/15) to 93% (14/15), in leadership from 13% (2/15) to 27% (4/15). In terms of health knowledge, excellent scores increased from 40% for leishmaniasis, 60% for malaria, and 13% for tuberculosis, reaching 80% for all three diseases. The qualitative evaluation showed positive results in terms of the participants’ perceptions of both the methodology and the co-design process outcomes.

**Conclusions:**

The co-design process was driven by 3 key factors: (1) active community participation at every stage; (2) knowledge exchange between multidisciplinary technical expertise and practical local knowledge; and (3) the use of innovative, culturally adapted pedagogical tools tailored to the rural context and population. This co-design process proved to be an effective method for meaningful capacity building among populations experiencing vulnerability in complex settings, and has the potential to contribute significantly to the improvement of infectious disease prevention and control.

## Introduction

### Background

Infectious diseases represent a public health challenge worldwide, particularly in low- and middle-income countries where their impact is most severe [1]. Participatory education is key in preventing and controlling infectious diseases because it enhances knowledge, raises awareness, and empowers communities [2]. Co-design, understood as a collaborative approach where various stakeholders, including community members, contribute to the development of interventions tailored to local contexts, also plays a crucial role in health strategies by improving health care access, reducing costs, and promoting local ownership [3-5].

This study was conducted in Pueblo Rico, a municipality in the department of Risaralda, Colombia, situated in a vast rainforest area endemic to tropical diseases [6]. Despite ongoing efforts by health institutions, infectious diseases remain a significant public health challenge in Pueblo Rico [1]. In 2022, the number of malaria cases surged to 1971; tuberculosis cases reached their highest level in 15 years, with 24 cases and an incidence rate of 113.5 per 100,000 inhabitants; and the number of cases of cutaneous leishmaniasis rose to 48 [7].

The populations mainly affected by infectious diseases are those living in rural areas, of whom 31% are Indigenous people from the Embera community, and 15.1% are people of Afro-Colombian descent [8]. All face a multidimensional poverty index of 82% [8], which heightens their risk for infectious diseases. Factors related to poverty, such as inadequate water management, poor sanitation, and overcrowding, significantly contribute to the disease burden in these areas [9]. Furthermore, the enduring impact of the armed conflict has left 5699 affected individuals in Pueblo Rico, including Embera communities recognized as eligible for collective reparations [10]. In previous studies, we have identified significant barriers to health care accessibility, limited facilities, and administrative issues within the Colombian health system [11]. In addition, low schooling levels, communication challenges, cultural conflicts between traditional and western medicine, and community mistrust of health personnel hinder effective health literacy and health-related behaviors of Embera populations [12].

We considered the characteristics of the rural population when culturally adapting the participatory audiovisual methods used in this study. Cultural adaptation refers to modifying or developing interventions to better align with the sociocultural characteristics and needs of a target population, in this case, ensuring comprehensive health education and promoting behavioral change to improve uptake, acceptance, and ultimately health outcomes [12,13]. Techniques such as dramatizations, drawings, photographs, and videos not only capture participants’ knowledge, experiences, and perspectives but also transcend language and literacy barriers, simplifying complex health concepts such as infectious disease transmission and prevention [14].

By fostering discussion and community involvement in content creation, participatory methods enhance engagement, ownership, and practical application of knowledge, making them particularly valuable in rural and ethnic communities with limited formal education and structural barriers [14]. The World Health Organization has promoted participatory approaches such as ENGAGE-TB, which emphasizes the importance of community involvement and participatory methods to enhance the reach and sustainability of tuberculosis services [6].

### Objectives

This study aims to describe the co-design process and evaluate the capacity building of an education program for the prevention and control of infectious diseases using participatory audiovisual methods culturally adapted to ethnic communities and rural contexts in Colombia.

## Methods

### Study Design

This paper presents the second phase of an implementation research project designed to enhance the prevention and control of malaria, tuberculosis, and leishmaniasis in Pueblo Rico through culturally adapted interventions. The first phase involved a participatory social diagnosis to identify barriers and facilitators to disease prevention and control. The second phase, explored in this paper, focuses on the co-design of a health education program, encompassing the training process and the cocreation of workshops and audiovisual materials (Table 1). The third and final phase will involve the program’s implementation.

A qualitative case study methodology was used for its exploratory and explanatory potential in open systems where context cannot be controlled [14,15]. Case studies are widely used in social innovation research to assess the effectiveness of social and cultural strategies [16].

**Table 1:** Co-design process.

Variables	Description	Program details
Training	Content Community work (leadership and pedagogy) Infectious diseases (malaria, leishmaniasis, and tuberculosis)	Sessions: 19 Duration: 160 h Month and year: September 2023
Workshop cocreation	Result: 12 theoretical-practical workshops 4 for malaria 4 for leishmaniasis 4 for tuberculosis	Sessions: 20 Duration: 120 h Month and year: October 2023
Audiovisual material cocreation	Result: 3 audiovisual products, each consisting of 4 episodes Stop-motion animation about malaria Comic book about leishmaniasis Puppet show about tuberculosis	Sessions: 20 Duration: 120 h Month and year: November 2023

### Participants

The co-design process involved 15 community leaders hired by the project to contribute to the design, implementation, and evaluation of the program. Participants were selected through convenience sampling, with support from social organizations and local authorities. Eligibility criteria included being aged >18 years; residing in Pueblo Rico for at least 10 years; speaking Spanish; being literate; and having experience, interest, or knowledge in health.

### Data Collection

For the training plan, workshops, and cocreated audiovisual materials, technical consultations with experts in malaria, leishmaniasis, and tuberculosis were conducted using nominal group exercises to identify key workshop topics. Simultaneously, a participatory social diagnosis was carried out to identify unhealthy practices, knowledge gaps, and negative attitudes, shaping the workshop objectives. Both techniques were led by the research team, after which an ethnoeducator developed the pedagogical design for the cocreation sessions.

The cocreation of workshops and audiovisual materials occurred through theoretical-practical sessions using participatory audiovisual methods. This process was made possible by the collaboration of multiple stakeholders: the research team, which guided content and methodology; the community leaders, who designed the workshops and contributed to audiovisual creation; and the audiovisual production team, which provided technical support. A total of 40 six-hour sessions were conducted (20 for workshop design and 20 for audiovisual production).

The evaluation focused on community leaders’ perspectives to understand their experiences and learning during cocreation. Qualitative data were collected through 2 focus groups, each lasting approximately two-and-a-half hours and conducted by the first author (MMB-G). The first focus group took place at the end of the training phase, after participants were introduced to theoretical concepts, while the second was held at the conclusion of the co-design process, emphasizing practical application. Both assessments examined perception, pedagogy, learning, skills, and critical thinking (Multimedia Appendix 1).

In addition, a quantitative evaluation of knowledge acquisition was conducted at the beginning and end of the co-design process by the research team. Individual initial and final assessments measured theoretical knowledge through 6 multiple-choice questions with images to aid comprehension, along with 2 open-ended questions for further exploration. The final evaluation also included a group exercise to assess participants’ acquired competencies and their ability to apply theoretical knowledge in practice. For the 3 infectious diseases under study, the evaluation covered disease overview, transmission cycles, diagnosis and treatment, and preventive behaviors. Leadership assessment focused on negotiation skills, teamwork, and communication, while pedagogy evaluation considered learning objectives, content structuring, practical application, and assessment criteria.

### Coding and Analysis

Focus groups were audio recorded, transcribed verbatim, and coded using ATLAS.ti software (Lumivero, LLC) by the second author (LSZ). Content analysis was conducted by MMB-G, considering both the learning process and participants’ perceptions of methodology. Quantitative data from multiple-choice questionnaires were manually coded, with scores weighted on a 5-point scale for individual evaluations. Final individual and group evaluation scores were averaged. Open-ended responses were scored based on their alignment with the correct answer. Evaluation data were categorized into 3 performance levels (low, medium, and high), with percentage values assigned to each. A comparative analysis was then performed to assess changes in knowledge by comparing the percentage distribution of scores from the initial and final evaluations.

### Ethical Considerations

The research was approved by the research ethics committee of Centro Internacional de Entrenamiento e Investigaciones Médicas (International Center for Training and Medical Research; 1272). To conduct this study, written informed consent was obtained from all participants involved. They are preserved in the physical and digital records of the project, which are for the exclusive use of the research team.

## Results

### Participants

Of the 15 participants, 9 (60%) were Indigenous people from the Embera community, and 6 (40%) were people of Afro-Colombian descent; moreover, 11 (73%) were women, and 4 (27%) were men. The participants were aged between 19 and 50 years. Of the 15 participants, 4 (27%) were nursing assistants, 4 (27%) were education technicians, 3 (20%) were high school graduates, 3 (20%) studied public health, and 1 (7%) was a psychologist.

### The Cocreation Process

**Figure 1:**
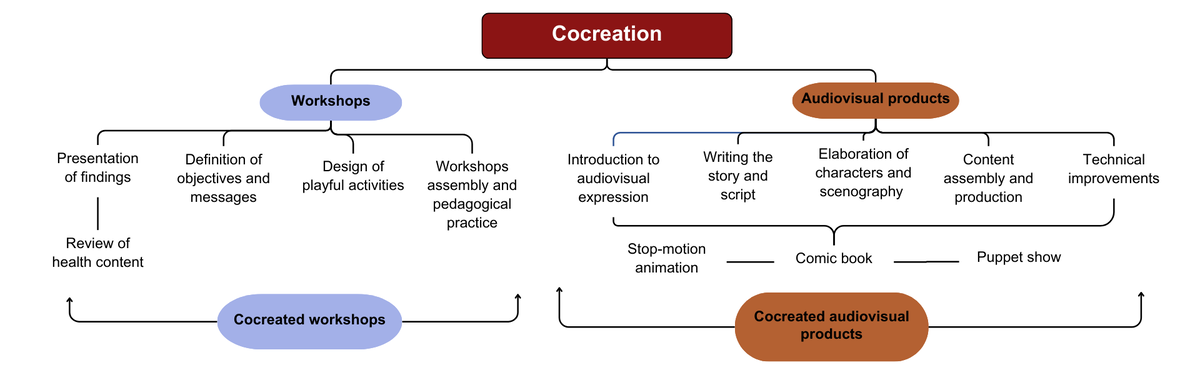
The cocreation process.

The cocreation process yielded 2 main outcomes (Figure 1). The first was the development of 12 workshops co-designed with the community leaders, with 4 workshops dedicated to each of the 3 diseases under study: leishmaniasis, malaria, and tuberculosis. Each workshop focused on a general theme: awareness and motivation, promotion of preventive practices, promotion of early diagnosis and timely treatment, and mitigation of risk factors. The community leaders were divided into 4 subteams, each responsible for designing 1 workshop for each disease.

The methodology for designing each workshop included 4 main steps. The first was preparation, which involved the presentation of the findings of the participatory diagnosis by the social research team, highlighting the strengths and weaknesses of the community regarding disease-related knowledge and behaviors. Basic information regarding the diseases was then reviewed with a health expert. In the second step, the social research and pedagogy teams collaborated with the community leaders to define a clear objective and message for each workshop. In the third step, playful activities for the workshops were designed by community leaders with the support of the social research and pedagogy teams: one to promote reflection, one to demonstrate learning, and one to promote action. Finally, in the fourth step, the community leaders reviewed and assembled the workshops they had designed and conducted a pedagogical practice where they shared the complete workshop with their peers.

The second main outcome of the cocreation process was the creation of 3 audiovisual products (refer to Multimedia Appendices 2-4): an animation about malaria [17], a comic book about leishmaniasis [18], and a puppet show about tuberculosis [19]. Each audiovisual product consisted of 4 episodes. The cocreation process involved the community leaders, audiovisual producers, the social research team, and a health expert who helped define themes and content. This process included 5 steps. The first step was an introduction to audiovisual expression, which included exercises such as dance to engage the creative side of the community leaders, as well as basic training in artistic techniques. The second step was defining the story and script, incorporating key and precise knowledge about the diseases under study. Next came the elaboration of characters, scenography, and other elements through drawing, painting, and other crafts. The fourth step involved assembly for the puppet show and recording for the animation. Finally, the audiovisual producers made technical adjustments and improvements to the products cocreated with the community leaders.

Once the audiovisual products were incorporated into the workshops, the final outcome was 12 workshops, each with five sections: (1) introduction of the workshop facilitators and main theme and a playful activity to determine preexisting knowledge about the theme; (2) content presentation, featuring 1 episode of the cocreated audiovisual material to explain the theme of the workshop and a presentation by the community leaders to elaborate on the theme; (3) a playful activity to practice what was learned; (4) a motivational activity to promote application in participants’ day-to-day lives; and (5) an evaluation of what participants learned and their perceptions of the workshop.

### Quantitative Evaluation

**Figure 2:**
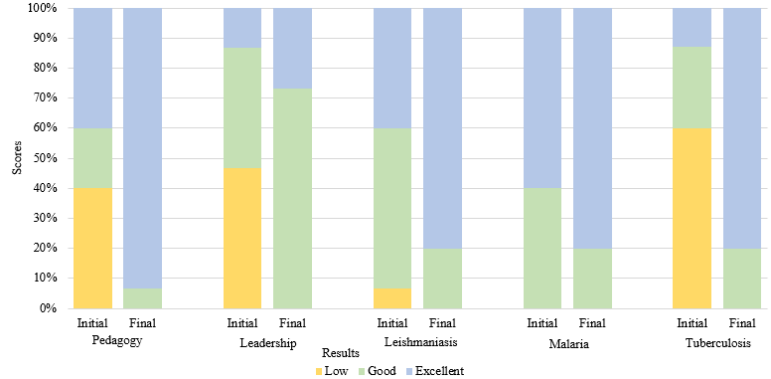
Quantitative evaluation results.

The quantitative evaluation (Figure 2) yielded positive outcomes for knowledge acquisition and significant improvement in leadership and pedagogy. In the initial evaluation, of the 15 participants, 7 (47%) demonstrated low leadership performance, and 6 (40%) showed low pedagogical performance; however, by the final evaluation, no participant scored low in either domain. For leadership, the proportion of participants with good results increased from 40% (6/15) to 73% (11/15), and the proportion of those with excellent results increased from 13% (2/15) to 27% (4/15). For pedagogy, the majority of the participants made substantial progress, with 93% (14/15) attaining excellent scores in the final evaluation.

Concerning health knowledge, specifically regarding leishmaniasis, the proportion of participants achieving excellent scores doubled, increasing from 40% (6/15) in the initial evaluation to 80% (12/15) in the final evaluation. Moreover, the elimination of low scores in the final evaluation represented notable progress. For malaria, participants already performed well in the initial evaluation, with no low scores and 60% (9/15) achieving excellent scores. By the final evaluation, the proportion with excellent scores increased to 12 (80%), reflecting a positive results. In contrast to malaria, tuberculosis presented the poorest initial scores, with 60% (9/15) of the participants attaining low scores and only 13% (2/15) achieving excellent scores. However, tuberculosis demonstrated the most significant improvement, with low scores being completely eliminated by the final evaluation. The proportion of participants with excellent scores surged to 80% (12/15), showcasing a substantial increase.

### Qualitative Evaluation

In the qualitative evaluation, one of the sections discussed participants’ perceptions of the methodology (Textbox 1), which were positive overall.

**Results from the coding process of the qualitative evaluation, summarizing participants’ perceptions of the methodology.**

**Learning facilitators**

- Clarity and precision (low concentration)
- Use of playful activities (low concentration)
- Use of humor (low concentration)
- Exchange of experiences (low concentration)

**Cultural exchange**

- Cultural practices (high concentration)
- Knowledge (medium concentration)
- Exchange (medium concentration)

**Evaluation**

- Innovative methodology (low concentration)
- Challenges (low concentration)

Participants emphasized pedagogical strategies that served as learning facilitators**.** These included the use of playful activities and humor in addressing pedagogy topics, as well as clarity, precision, and constant reiteration when discussing health-related topics. In addition, drawing on community health work experiences in similar contexts from different parts of the world as inspirational examples was recognized as a valuable strategy to motivate participants. Both Embera and Afro-Colombian participants emphasized the value of having a mixed group with members of both communities because through the cultural exchange, they learned about each other’s cultural practices, and it allowed the Embera participants to develop new language skills. Finally, the community leaders discussed the evaluation, noting that performing group evaluations allowed each participant to contribute what they knew and that including traditional elements from their culture in the evaluation was a novel approach. However, they also identified challenges in terms of time management and understanding the evaluations:

We spent time together with the Afros today, we had fun, if we didn’t understand a word, we learned from them. I didn’t know too many words, but with them we learned a little bit, now I understand more.

Embera participant; focus group; November 2023

The second section of the qualitative evaluation (Table 2) centered on the learning process, assessing participants’ performance in 6 focus areas (the 3 diseases under study and the domains of leadership, pedagogy, and audiovisual creation). For the diseases, the initial evaluation revealed new learning identified by participants, which increased by the final evaluation. This improvement was evidenced in the greater quantity, specificity, and detail of the responses and topics mentioned.

**Table 2:** Results from the coding process of the qualitative evaluation, summarizing participants’ perceptions of learning related to the focus areas (diseases and domains).

Focus areas			Initial evaluation		Final evaluation
**Diseases**					
	Leishmaniasis	Vector characteristics^a^ Transmission cycle^a^		Prevention strategies^a^ Vector characteristics^a^ Treatment adherence^a^ Timely diagnosis^a^ Types of leishmaniasis^a^ Importance of balanced diet^a^	
	Malaria	Treatment^b^ Prevention strategies^a^ Transmission cycle^a^ Vector characteristics^a^		Timely diagnosis^a^ Treatment adherence^a^ Importance of going to the physician^a^ Prevention strategies^a^ No self-medication^a^ Importance of balanced diet^a^	
	Tuberculosis	Prevention strategies^a^ Symptoms^a^ Treatment^a^		Symptoms^b^ Transmission^a^ Prevention strategies^a^ Timely diagnosis^a^ Importance of balanced diet^a^	
**Domains**					
	Leadership	Characteristics of a leader^b^ Experiences of world leaders^a^ Difficulties of dealing with a new subject^a^		Public speaking^c^ Characteristics of a leader^a^	
	Pedagogy	Methodologies adequate for the context^a^ Learn by teaching^a^ Crafting objectives^a^ Planninga		Crafting objectives^b^ Writing messages^a^ Importance of planning^a^ Describing activities step by step^a^	
	Audiovisual creation	Development of the product^b^ Usefulness^a^		Audiovisual techniques^c^ Crafts^c^ Usefulness^b^ Technology^a^	

^a^Low concentration.

^b^Medium concentration.

^c^High concentration.

Concerning leishmaniasis, the initial evaluation showed that participants primarily learned about vector characteristics and the transmission cycle. In the final evaluation, there was a notable improvement, with the participants demonstrating knowledge not only about vector characteristics but also about prevention strategies, the importance of treatment adherence and timely diagnosis, the types of leishmaniasis, and the importance of a balanced diet. An example of the knowledge acquired about leishmaniasis is illustrated in the following quote:

When they get the medicines, they should have the entire treatment applied and not interrupt the application, because if they interrupt it, the parasite is not going to die and then they are going to get more lesions in other places.

Afro-Colombian participant; focus group; November 2023

In the case of malaria, the initial evaluation had the highest number of responses and topics mentioned, covering vector characteristics, prevention strategies, transmission cycle, and treatment. However, as with the other diseases, there was an increase in the specificity of the responses in the final evaluation, with participants additionally mentioning timely diagnosis, treatment adherence, the importance of seeking medical attention and not self-medicating, and the importance of a balanced diet. A participant stated as follows:

In malaria, it is important to finish the treatment so that the bug that enters our body dies, it’s completely eradicated, because if we take the first four, five days, we feel relieved, and we abandon the treatment, then the disease will get worse.

Afro-Colombian participant; focus group; November 2023

Regarding tuberculosis, in the initial evaluation, participants mainly mentioned learning about prevention strategies, symptoms, and treatment. There was an improvement in the final evaluation, in which they reiterated learning about prevention strategies and symptoms while adding learning about transmission, timely diagnosis, and the importance of a balanced diet:

[A] mother always waits for 15 days when children have the flu, “oh, it’s a normal flu,” but you don’t know if it is tuberculosis, so go to the hospital in time to find out if it is tuberculosis, you have to go to the hospital.

Embera participant; focus group; November 2023

With regard to the domain of leadership, in the initial evaluation, participants noted their acquisition of theoretical knowledge, such as the characteristics of a leader, as well as insight into the experiences of renowned world leaders. At the same time, they recognized that it was a difficult subject because it was new to them. However, in the final evaluation, participants highlighted the development of practical public speaking skills:

For my part, I participated for two months and I improved a lot, talking in public during the presentations I have done to give the messages...for my part, that improved everything, my shyness, at the beginning I was very shy, but little by little I improved, I stopped being shy.

Embera participant; focus group; November 2023

Regarding the pedagogy domain, in the initial evaluation, participants highlighted learning about the importance of using the appropriate methodology to reach their communities, including the use of audiovisual tools and playful activities. However, they encountered difficulties in grasping more practical aspects, such as planning and crafting objectives for the workshops. These challenges persisted in the final evaluation; for instance, they mentioned the challenges in describing activities step by step. Nonetheless, they recognized the importance of planning to better address community needs, establishing clear objectives to guide workshops, and creating clear messages without using technical terms to facilitate the community’s comprehension of the topics:

It’s just like a necklace [talking about planning], the necklace when you are crafting it is the same.... I have always made necklaces and when you don’t start well, then it gets tangled up and that’s how it ends, it stays tangled up and it doesn’t look good.

Embera participant; focus group; November 2023

The last domain evaluated was audiovisual creation. In the initial evaluation, participants had only engaged in the creation of basic audiovisual products during the workshops. Despite their limited experience, they expressed their enjoyment in developing this type of product, particularly highlighting TikTok videos and radio dramas, with a participant noting the usefulness of audiovisual products for community education.

By contrast, in the final evaluation, they emphasized the value they found in learning to create diverse types of audiovisual products, especially animations. However, they reported facing several challenges during the creation process, such as experiencing frustration with the time-consuming nature of stop motion or the physical demands of assembling a puppet show. These challenges, common when working with artistic or physical skills, did not hinder the process. Instead, they were acknowledged and mitigated by balancing activities during implementation.

Another aspect participants emphasized was the enjoyment they found in crafting visual elements for the audiovisual products, such as creating drawings for comics and crafting puppets for shows. They also noted improvement in their artistic skills. Furthermore, they found the use of apps to be an interesting aspect of the process; however, they encountered technological barriers. Regarding the utility of the products, participants emphasized their potential to amplify the impact of the workshops by reaching more people due to their participatory nature, which facilitates engagement and learning, as expressed by a participant:

Yes, the comics would be good for coloring. It would be good because they are going to be entertained and they are going to gain knowledge about the mosquito, the dog....

Afro-Colombian participant; focus group; November 2023

## Discussion

### Principal Findings

The co-design process showed that participatory methods, knowledge exchange, and culturally adapted tools enhanced ownership, engagement, and knowledge acquisition, leading to significant improvements and positive feedback.

#### Key Factors of Co-Design Useful for Replication

Throughout the development and evaluation of this co-design process, 3 key factors were identified as useful for replication in future studies, based on both the research team’s experience and the results obtained from the qualitative evaluation, including participants’ perceptions of the methodology, learning facilitators, cultural exchange, and evaluation. The first factor was the participatory approach used throughout the process (diagnosis, design, and implementation). As evidenced in other studies [20], this approach empowers participants to develop a sense of joint ownership over the project, helps to build trust between the participants and the research team, and facilitates the integration of research into practice [21]. In this study, applying the participatory approach at every stage led to participants seeing their contributions reflected in the strategies and co-designed audiovisual products, giving them a stake in the project’s success and facilitating the next phase, which involves implementing these strategies with the community at large.

The second factor was the knowledge exchange process involving multiple stakeholders: community leaders, who contributed expertise based on their lived experience; social researchers, who brought expert knowledge of community work and pedagogy; a health expert, who contributed expertise on infectious diseases; and audiovisual producers, who provided technical knowledge on audiovisual production. In line with the literature [22], to ensure the success of the co-design process, the researchers acted as facilitators who promoted capacity building and provided tools and methodological structures [23] to support the community leaders in creating workshops and audiovisual products. This process acknowledged the different levels of interest, creativity, and skills among the community leaders. The knowledge exchange process enabled researchers to understand the actual conditions experienced by the community and learn how to make interventions feasible, while also equipping the community leaders with tools to act within their own context. Diversity among participants, in terms of ethnicity, gender, educational level, and health knowledge, and the inclusion of representatives of health workers as well as community members who are beneficiaries of health interventions, played an important role. Within the research team, interdisciplinarity was key.

The third and final factor was the use of innovative, culturally adapted pedagogical tools. Throughout the co-design process, creative strategies such as the use of digital and audiovisual tools, case studies set in similar contexts, and games and playful activities were involved in the facilitation of knowledge acquisition. To be effective, these strategies required adaptation to better respond to the context and cultural characteristics of the population, and they were tied to attempts to integrate the cultural traditions of the communities involved into the co-design process, which enhanced their acceptability among the participants.

#### Capacity Building for Ownership

The Design Council of the United Kingdom defines “co-design” as “the meaningful involvement of end users in the design process” [24]. In this study, co-design with end users helped develop the skills and knowledge necessary for achieving ownership of interventions aimed at improving community health conditions. One of the most important accomplishments of this cocreation process was building capacity within the community and promoting meaningful learning through a theoretical-practical methodology that enabled effective training and helped overcome barriers related to low schooling levels and communication. The co-design process evaluated in this study involved training in health topics and skills for community work, as well as the cocreation of workshops and audiovisual products. The quantitative evaluation showed positive results regarding knowledge acquisition by the community leaders, and the qualitative evaluation demonstrated positive perceptions of the methodology and the learning outcomes. Consistent with previous studies [25], the involvement of the community leaders in creating audiovisual materials, as well as the use of traditional games and playful activities, facilitated the presentation and explanation of complex information, the improvement of comprehension and recall, and the promotion of engagement and skill development. These benefits were recognized by the community leaders in their qualitative evaluations, and the positive outcomes were also reflected in the quantitative results.

#### Contribution to Health Outcomes

Community participation was recognized in the Declaration of Alma-Ata as essential for primary health care [26], and diverse studies have shown its contribution to the prevention and control of infectious diseases. The co-design process can be understood as participative, enabling better understanding of context and background, while scientific knowledge enhances and supports the design of evidence-based solutions to improve health conditions in communities experiencing vulnerability that are affected by infectious diseases [27].

In this study, the cultural adaptation of content was crucial to better respond to participants’ literacy levels, communication barriers, and identified skills. As shown in previous studies [28,29], culturally adapting health education to respond to such population characteristics improves its effectiveness. In this case, the cultural adaptation involved presenting clear and precise information, constantly repeating information, incorporating playful activities and audiovisual materials, and using examples of community health workers in similar contexts. Participants evaluated these strategies positively in the qualitative assessment, and the quantitative results showed marked improvement in knowledge acquisition across the 6 focus areas (diseases and domains) addressed.

Furthermore, the participation of the community leaders in the creation process allowed the audiovisual products to be better adapted to the context and population characteristics because their preferences could be incorporated from the outset in a more meaningful way than if they had been involved in the adaptation only after the initial products had been already created, an approach that is also in line with findings from other studies [28]. As a result of the cocreation process, the audiovisual products conveyed clear messages using simple language and familiar images; incorporated colloquialisms and idioms; featured Embera and Afro-Colombian characters; and reflected community settings and cultural practices, including traditional medicine.

### Limitations

As a case study, this research prioritized depth over representation; accordingly, a purposive sample was selected. In future studies, the development of other case studies will be useful for comparing and generalizing the findings of this study.

### Conclusions

The co-design process was driven by three key factors: (1) active community participation at every stage; (2) knowledge exchange between multidisciplinary technical expertise and practical local knowledge; and (3) the use of innovative, culturally adapted pedagogical tools tailored to the rural context and population. This co-design process proved to be an effective method for meaningful capacity building among populations experiencing vulnerability in complex settings and has the potential to contribute significantly to the improvement of infectious disease prevention and control.

## Appendix

Multimedia Appendix 1 Focus groups guide.

Multimedia Appendix 2 Co-designed audiovisual product (a comic book) about cutaneous leishmaniasis.

Multimedia Appendix 3 Co-designed audiovisual product (a puppet show) about tuberculosis.

Multimedia Appendix 4 Co-designed audiovisual product (an animation) about malaria.
