# Supplementary material for: Developing a Health Education Program for the Prevention and Control of Infectious Diseases Culturally Adapted to Ethnic and Rural Communities: Co-Design Study Using Participatory Audiovisual Methods
Source: J Particip Med. 2025 Aug 29;17:e65116. doi: 10.2196/65116 (PMC12432463; doi:10.2196/65116)
Supplement: Multimedia Appendix 1 [file jopm_v17i1e65116_app1.docx]

**FOCUS GROUP GUIDE IMPLEMENTATION PHASE**

**OBJECTIVE:** Evaluate the process of social appropiation of knowledge and significant learning of the Training Plan developed during the month of September with an intensity of 160 hours.

**Questions**

**General Perception**

1. What do you think of the activities we have done this month?
2. What did you like the most and the least?
3. How did you like the experience of sharing 6 hours a day with your classmates and the facilitators?

**Pedagogy**

1. How did it feel to have a full month of workshops or training? How did it feel to go back to study?
2. What has been easy and what has been difficult?
3. Have you seen any differences from other trainings or workshops you have participated in? Which ones?

**Learning**

1. What new things (that you didn’t know before) have you learned?
2. Did you realize that some of the things you thought about diseases were not correct?
3. Do you remember whih were the 5 modules we worked on? Which was the module you liked the most? Why?
4. Which activities do you remember? Which one did you like the most? Why?
5. What do you remember about the evaluation?

**Skills development**

1. What thibgs from the workshops can be useful for being a health leader?
2. Have you learned anything new to be a health leader? What do you still need to improve?
3. How do you see yourself as a health leader in the future?

**Critical Thought**

1. Do you recall any examples of health interventions that have caught your attention, any that could perhaps be replicated in Pueblo Rico?
2. How do you consider the infectiou disease management situation in Pueblo Rico?
3. Do you think it is posible for Pueblo Rico’s health situation in Malaria, Leishmaniasis and Tuberculosis to improve? What is the key?
